# New Radiomic Markers of Pulmonary Vein Morphology Associated With Post-Ablation Recurrence of Atrial Fibrillation

**DOI:** 10.1109/JTEHM.2021.3134160

**Published:** 2021-12-09

**Authors:** Michael A. Labarbera, Thomas Atta-Fosu, Albert K. Feeny, Marjan Firouznia, Meghan Mchale, Catherine Cantlay, Tyler Roach, Alexis Axtell, Paul Schoenhagen, John Barnard, Jonathan D. Smith, David R. Van Wagoner, Anant Madabhushi, Mina K. Chung

**Affiliations:** Cleveland Clinic Lerner College of MedicineCase Western Reserve University 2546 Cleveland OH 44106 USA; Department of Biomedical EngineeringCase Western Reserve University 2546 Cleveland OH 44106 USA; Department of Cardiovascular and Metabolic SciencesLerner Research Institute, Cleveland Clinic Cleveland OH 44106 USA; Department of Cardiovascular Medicine, Heart, VascularThoracic Institute, Cleveland Clinic Cleveland OH 44106 USA; Department of Quantitative Health SciencesLerner Research Institute, Cleveland Clinic Cleveland OH 44106 USA; Louis Stokes Cleveland Veterans Administration Medical Center Cleveland OH 44106 USA

**Keywords:** Cardiology, electrophysiology, biomedical imaging, machine learning, biomarkers

## Abstract

*Objective:* To identify radiomic and clinical features associated with post-ablation recurrence of AF, given that cardiac morphologic changes are associated with persistent atrial fibrillation (AF), and initiating triggers of AF often arise from the pulmonary veins which are targeted in ablation. *Methods:* Subjects with pre-ablation contrast CT scans prior to first-time catheter ablation for AF between 2014–2016 were retrospectively identified. A training dataset (D_1_) was constructed from left atrial and pulmonary vein morphometric features extracted from equal numbers of consecutively included subjects with and without AF recurrence determined at 1 year. The top-performing combination of feature selection and classifier methods based on C-statistic was evaluated on a validation dataset (D_2_), composed of subjects retrospectively identified between 2005–2010. Clinical models ( $\text{M}_{\mathrm {C}}$) were similarly evaluated and compared to radiomic ( $\text{M}_{\mathrm {R}}$) and radiomic-clinical models ( $\text{M}_{\mathrm {RC}}$), each independently validated on D_2_. *Results:* Of 150 subjects in D_1_, 108 received radiofrequency ablation and 42 received cryoballoon. Radiomic features of recurrence included greater right carina angle, reduced anterior-posterior atrial diameter, greater atrial volume normalized to height, and steeper right inferior pulmonary vein angle. Clinical features predicting recurrence included older age, greater BMI, hypertension, and warfarin use; apixaban use was associated with reduced recurrence. AF recurrence was predicted with radio-frequency ablation models on D_2_ subjects with C-statistics of 0.68, 0.63, and 0.70 for radiomic, clinical, and combined feature models, though these were not prognostic in patients treated with cryoballoon. *Conclusions:* Pulmonary vein morphology associated with increased likelihood of AF recurrence within 1 year of catheter ablation was identified on cardiac CT. *Significance:* Radiomic and clinical features-based predictive models may assist in identifying atrial fibrillation ablation candidates with greatest likelihood of successful outcome.

## I. Introduction

Atrial fibrillation (AF) is the most common sustained arrhythmia in clinical practice, affecting 1–2% of the population [1], with lifetime risk of developing AF increasing to 37% after age 55 [2]–[4]. AF increases risk of systemic thromboembolism such as transient ischemic attack (TIA) and stroke, as well as risk of cardiovascular death [5]. Initiation of AF typically results from extra-nodal electrical activation, most often from the pulmonary veins, which are the primary target of AF ablation [6]. However, comorbidities and AF itself can cause electrical and structural remodeling that limits success of pulmonary vein isolation (PVI) procedures. Thus recurrence rates are high [7] and identifying patients likely to have successful ablation outcomes and which anatomic substrates should be targeted remains controversial [8], [9].

Prior structural markers found to be associated with AF include pulmonary vein morphology [10], [11] and left atrial size [12]. Differences in pulmonary vein morphology and radiomic (computer extracted measurements) assessment of the left atrium have potential in screening candidates for ablation [13]–[18] or guiding scar-based ablation [19], [20]. CT radiomic, and/or magnetic resonance imaging (MRI) are often obtained prior to AF ablation to help guide anatomic localization of ablation substrates, including delineation of gross pulmonary vein morphology or determination of left atrial scarring via delayed enhancement on MRI. However, morphological left atrial or pulmonary vein features predictive of AF susceptibility or AF ablation outcome have not yet been well identified. Genetic variants associated with embryologic development of the pulmonary veins may influence AF ablation success via changes in pulmonary vein morphology.

**FIGURE 1.**
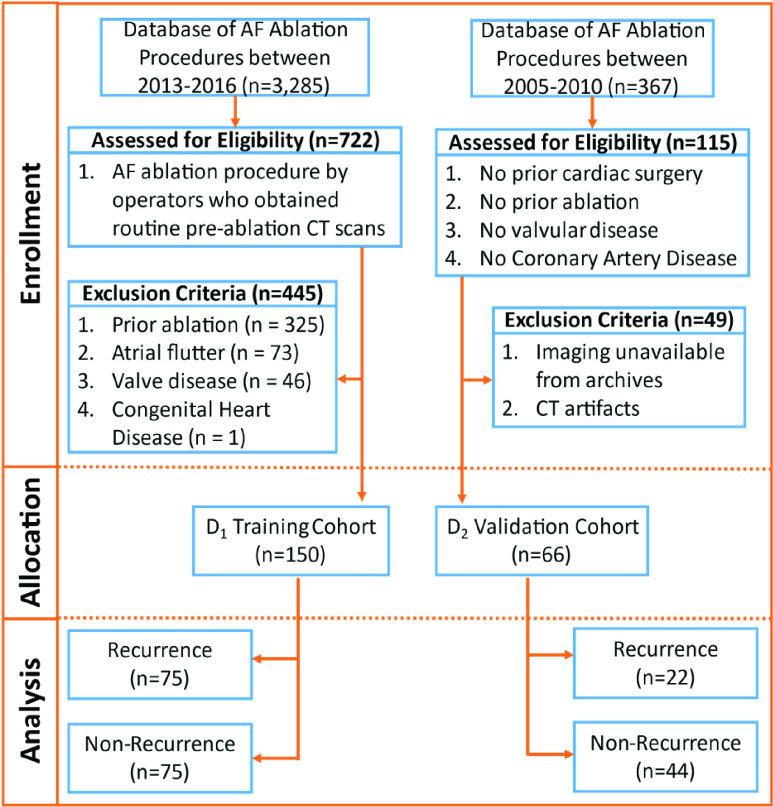
Summary of patient selection criteria for inclusion in this study.

The premise of this study was that new radiomic features relating to the pulmonary veins and left atrium from pre- ablation cardiac CT, in conjunction with clinical parameters (e.g. age, BMI) could help predict response to AF ablation. To evaluate this hypothesis, a machine classifier was constructed using the most distinguishing radiomic features associated with recurrence and validated on an independent cohort. Since clinical characteristics including age, sex, AF type and others have been associated with likelihood of post-ablation recurrence of AF, we constructed and independently validated machine classifier models using clinical features and compared their ability to predict recurrence with radiomic-based models, and hybrid radiomic-clinical models. An overview of our methodology is illustrated in Figure 1.

## II. Materials and Methods

### A. Data

Catheter ablation for AF has been previously described [13], [21]. Catheter access for AF ablation was obtained in the femoral vein and catheters advanced through the inferior vena cava and into the right atrium. The left atrium was accessed through trans-septal puncture or a patent foramen ovale, and pulmonary vein isolation was performed using radiofrequency or cryoballoon ablation [9], [22]. AF ablation success was assessed via the use of an in-house “AF tracker” system [23] at follow-up visits, in which the electrophysiologist utilizes a structured documentation format, and web-based patient survey at follow-up appointments of 3, 6, and 12 months post-ablation, as well as chart review. The study was approved by the Institutional Review Board for retrospective medical records review and performed in accordance with institutional guidelines. Training data, D_1_, were obtained via retrospective chart review of patients receiving AF ablation at Cleveland Clinic between July 2013 and November 2016 (Figure 1). Inclusion criteria included history of AF and cardiac CT imaging with contrast obtained prior to AF ablation. Only clinicians who routinely perform pre-ablation CT were selected to avoid selection bias for patients with other comorbidities warranting CT. In cases where multiple scans were available, the last acquired scan prior to ablation was selected (median 1 day prior to ablation, range 1–700 days). Subjects were excluded with prior AF ablation, cardiac surgery, congenital heart disease, or valve disease. From those who met these criteria (n=277), 150 were selected in chronological order of ablation by outcome of AF recurrence within 1 year post-PVI (n=85 excluded) to produce a balanced dataset for optimizing machine learning model training. Data for independent validation, D_2_, were similarly obtained via retrospective chart review of patients undergoing AF ablation at the same academic hospital between 2005 and 2010, in the absence of known structural heart disease or coronary artery disease (n=66, n=22 with recurrence of AF within 1 year). Recurrence of AF was determined by review of clinician assessment at 1 year post-PVI follow-up, exclusive of an initial 3-month blanking period.

### B. Annotation and Segmentation of Pulmonary Veins

Radiomic features of the left atrium and pulmonary veins were obtained (M.L., M.M., C.C., T.R., A.A., in-training) from pre-ablation, ECG-gated CT images with IV contrast enhancing the pulmonary veins (i.e. pulmonary vein protocol), using regimented measurement protocols designed with a board- certified cardiac imaging specialist (P.S.) in the Syngo.Via software suite (Siemens Healthineers USA). Pulmonary veins were identified based on contrast enhancement and cardiac anatomy vis-à-vis their insertion into the left atrium. Annotation was accomplished by computer-assisted image rotation and measurement, with cross-sectional pulmonary vein geometries obtained within 2cm from the PV-atrial junction and normal to the central axis of each vein. Images were segmented semi-autonomously using the AI-trained Syngo.Via three-dimensional segmentation tool, and manually refined to exclude contrast enhancement of the left ventricle and pulmonary veins further than 2cm from the PV-atrial junction. Segmentations were used to reliably measure left atrial volume for inclusion of volume-derived radiomic features into predictive models.

### C. Feature Extraction

A total of 119 features were extracted (40 radiomic and 79 clinical) including measurements describing left atrial morphology, pulmonary vein size, and incident angles of the pulmonary veins. Clinical data were obtained from a prospectively-collected clinical database maintained for all patients undergoing any cardiac electrophysiology procedure, and included demographic information, comorbidities, pertinent cardiac history, medications, and procedure data (supplemental materials, Table S1).

### D. Statistical Analysis

**FIGURE 2.**
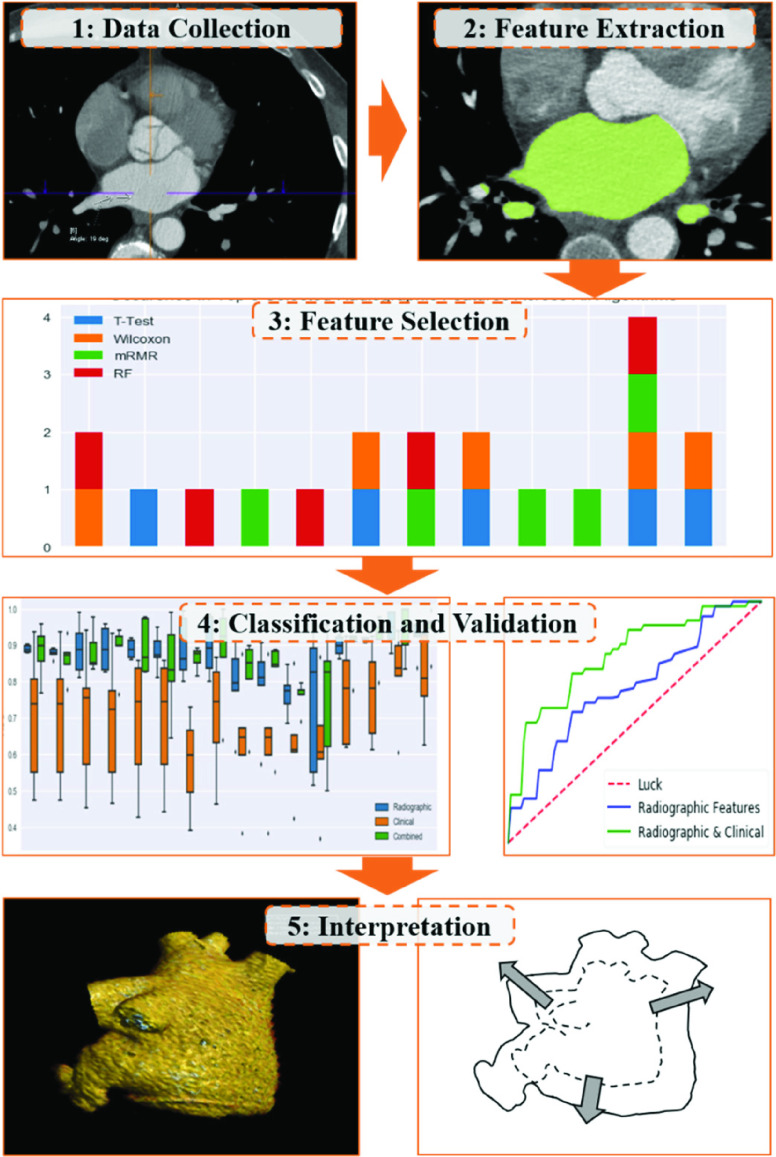
Overview of experiment methodology from data and radiomic feature extraction to selection, classification, and validation on an independent cohort.

Statistical analysis was executed using a platform built in- house on the Python programming language (v. 3.6.3) and packages including SciKit Learn and PymRMR (v. 0.1.8). Additional information regarding the software packages utilized is summarized in supplemental materials (Table S2). A pipeline consisting of sixteen combinations of four feature selection and four classifier algorithms were exhaustively applied to D_1_ to identify the reduced set of features and classifier that best predict the outcome of AF recurrence within 1 year post-PVI (Figure 2). Feature selection methods of t-test [24], Wilcoxon rank sums [25], minimum Redundancy Maximum Relevance (mRMR) [26] and random forest Gini importance [27] were applied to data preprocessed to a mean of 0 and unit variance. A 15:1 subject: feature metric was implemented to identify a reduced feature set of the top five distinguishing radiomic features in D_1_, and similarly for clinical features for comparison, and logistic regression was applied to quantify feature effects via odds ratios. Classifier models of linear discriminant analysis, quadratic discriminant analysis, support vector machines, and random forest, were trained on D_1_ top-selected features, and model performance was assessed by the area under the receiver operating characteristic curve (AUC) on the D_1_ training set with 5-fold cross-validation. Comparisons were made on this basis between models trained on radiomic features ( $\text{M}_{\mathrm {R}}$), clinical features ( $\text{M}_{\mathrm {C}}$), and a combination of radiomic and clinical features ( $\text{M}_{\mathrm {RC}}$) as trained on D_1_ (on all subjects, and by ablation and AF types) and validated on the entire independent set D_2_ with the SMOTE technique [28] applied to accommodate for outcome- imbalance (averaged over n=50 runs).

## III. Results

A summary of characteristics for all subjects included in this study is shown in Table 1. For training dataset (D_1_) subjects with recurrence at 1 year, greater magnitudes for CHA_2_DS_2_- VASc, BMI, and age were observed as compared to subjects without recurrence at 1 year. Within the recurrence cohort, there was also a greater proportion of diagnoses of hypertension and heart failure. No statistically significant differences were observed by outcome for the validation dataset (D_2_), though all D_2_ subjects underwent RF ablation having predated institutional cryoballoon use.

**TABLE 1:** Comparison of Clinical Characteristics by Recurrence of Atrial Fibrillation Within 1 Year of Catheter Ablation

Characteristic	D_1_ Training Set			D_2_ Test Set			
	No Recurrence at 1 Year	Recurrence at 1 Year	p-Value	No Recurrence at 1 Year	Recurrence at 1 Year	p-Value
No. of patients	75	75	-	44	22	-
RF Ablation	52 (69%)	56 (75%)	0.72	44 (100%)	22 (100%)	1.0
CHA_2_DS_2_-VASc	1.88 ± 1.5	2.88 ± 1.7	0.01	1.1 ± 1.0	1.2 ± 1.0	1.0
LVEF, %	55.1 ± 8.3	56.6 ± 8.5	0.38	54.9 ± 6.1	55.7 ± 7.2	1.0
Height, cm	178.2 ± 9.0	175.5 ± 10.6	0.20	179.2 ± 9.0	177.8 ± 13.0	1.0
Weight, kg	93.1 ± 19.3	97.6 ± 20.1	0.29	95.9 ± 16.6	96.6 ± 21.8	1.0
BMI, kg/m^2^	29.2 ± 5.1	31.6 ± 5.6	0.03	30.0 ± 5.7	30.6 ± 6.3	1.0
Age, years	62.1 ± 8.9	66.9 ± 8.7	0.01	57.1 ± 9.5	56.8 ± 10.7	1.0
LA Volume, cm^3^	153.4 ± 42.0	162.8 ±45.8	0.30	114.4 ± 30.0	129.7 ± 35.2	1.0
Male	59 (79%)	47 (63%)	0.12	37 (84%)	17 (77%)	1.0
Non-smoker	47 (63%)	51 (68%)	0.72	29 (66%)	15 (68%)	1.0
Paroxysmal AF	53 (71%)	40 (53%)	0.12	30 (68%)	10 (45%)	1.0
Diabetes	8 (11%)	18 (24%)	0.12	2 (5%)	2 (9%)	1.0
Hypertension	30 (40%)	49 (65%)	0.02	22 (50%)	10 (45%)	1.0
Heart Failure	21 (28%)	37 (49%)	0.05	2 (5%)	2 (9%)	1.0
CVA History	4 (5%)	1 (1%)	0.50	2 (5%)	1 (5%)	1.0
TIA History	3 (4%)	2 (3%)	1.00	1 (2%)	0 (0%)	-
Beta Blocker	43 (57%)	44 (59%)	1.00	37 (84%)	19 (86%)	1.0
AAD	51 (68%)	54 (72%)	0.80	41 (93%)	20 (91%)	1.0
ACE Inhibitor	13 (17%)	21 (28%)	0.29	2 (5%)	4 (18%)	1.0

Values are presented as mean ± SD or as n (%); p values corrected for multiple comparisons by False Detection Rate method RF =Radio-frequency; LVEF = left ventricular ejection fraction; BMI = body mass index; LA = left atrial; AF = atrial fibrillation; CVA = Cerebrovascular Accident; TIA = Transient Ischemic Attack; AAD = anti-arrhythmic drug; ACE =angiotensin-converting enzyme.

### A. Experiment 1: Radiomic Features Predict AF Recurrence Post-Ablation

**FIGURE 3.**
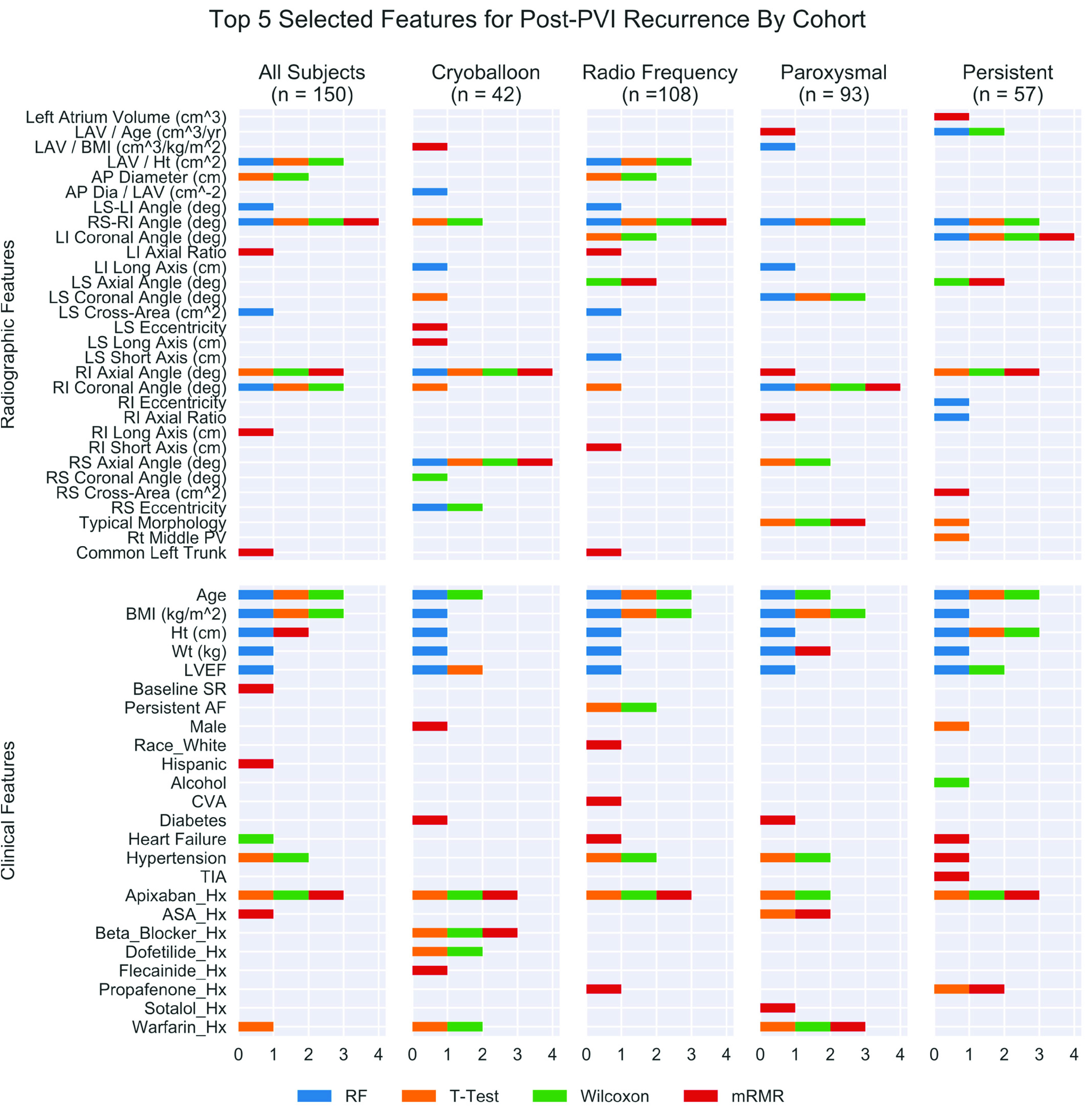
Summary of top five radiomic and top five clinical features each selected by method with 5-fold cross validation for all, radio-frequency, paroxysmal AF, and persistent AF subject (other features not selected are not represented). AP = Anterior Posterior; LAV = Left Atrial Volume; LI = Left Inferior; LS = Left Superior; RI = Right Inferior; RS = Right Superior; PV = Pulmonary Vein; Hx = History; Ht = Height; LVEF = Left Ventricular Ejection Fraction; AF = Atrial Fibrillation.

**FIGURE 4.**
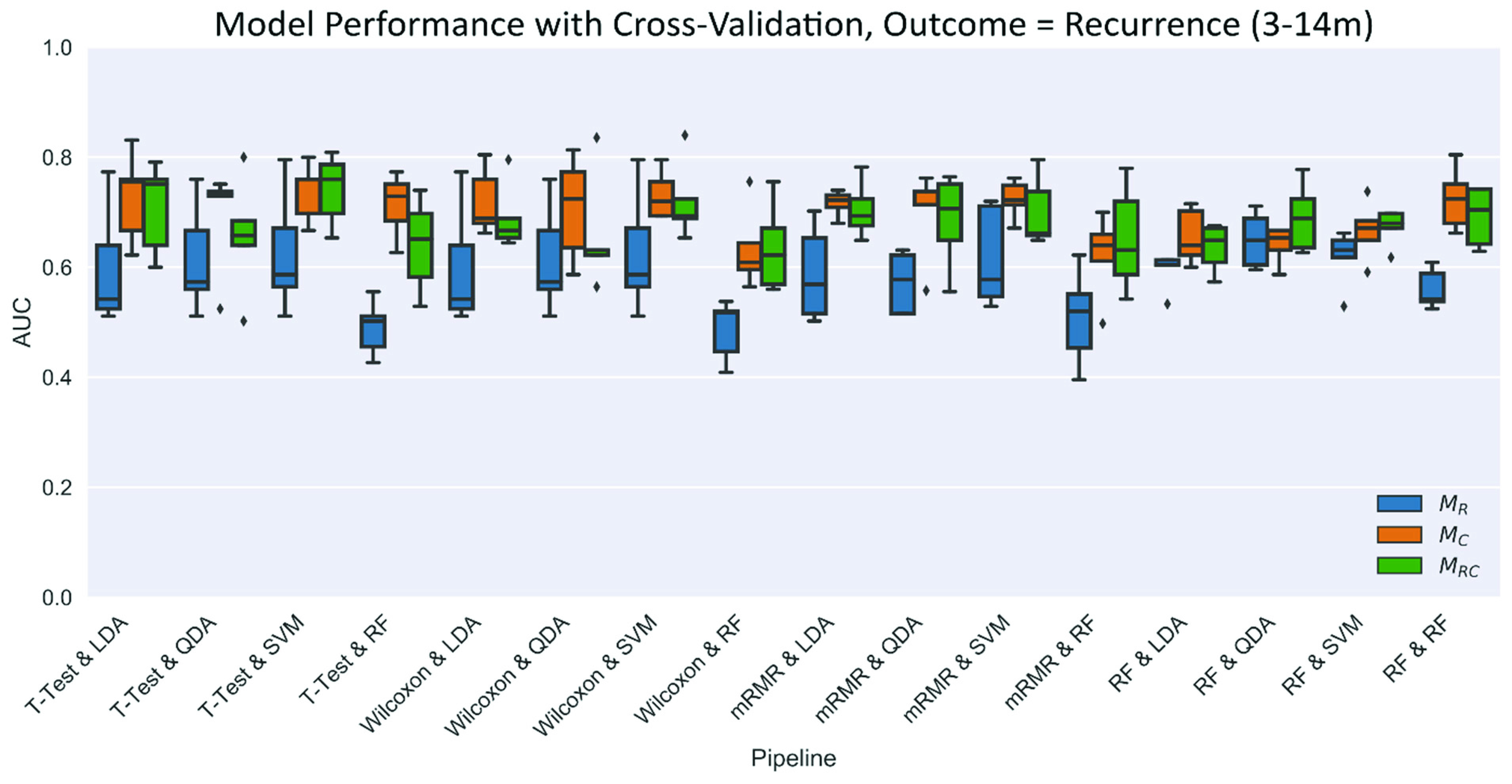
Classifier performance with cross-validation for post-PVI recurrence of AF within 1 year for D_1_ all subjects (n=150) with 16 combinations of feature selection and classifier models. Models were trained on radiomic features ( $\text{M}_{\mathrm {R}}$), clinical features ( $\text{M}_{\mathrm {C}}$), and the combination of radiomic and clinical features ( $\text{M}_{\mathrm {RC}}$).

The objective of this experiment was to demonstrate that features of pulmonary vein and atrium morphology predict post-ablation recurrence of AF. For the 40 radiomic and 79 clinical features considered, the five most-distinguishing radiomic and clinical features for recurrence of atrial fibrillation within 1 year of catheter ablation are summarized in Figure 3. When considering all subjects, four radiomic features were consistently identified in the top five measurements across all 16 pipelines: right carina angle, left atrial volume normalized to height, and entry angle of the right inferior pulmonary vein on the axial and coronal planes. Radiomic classifier ( $\text{M}_{\mathrm {R}}$) performance for each of the sixteen combinations of four feature selection and four classifier methods applied with cross-validation to all subjects in D_1_ are shown in Figure 4.

**FIGURE 5.**
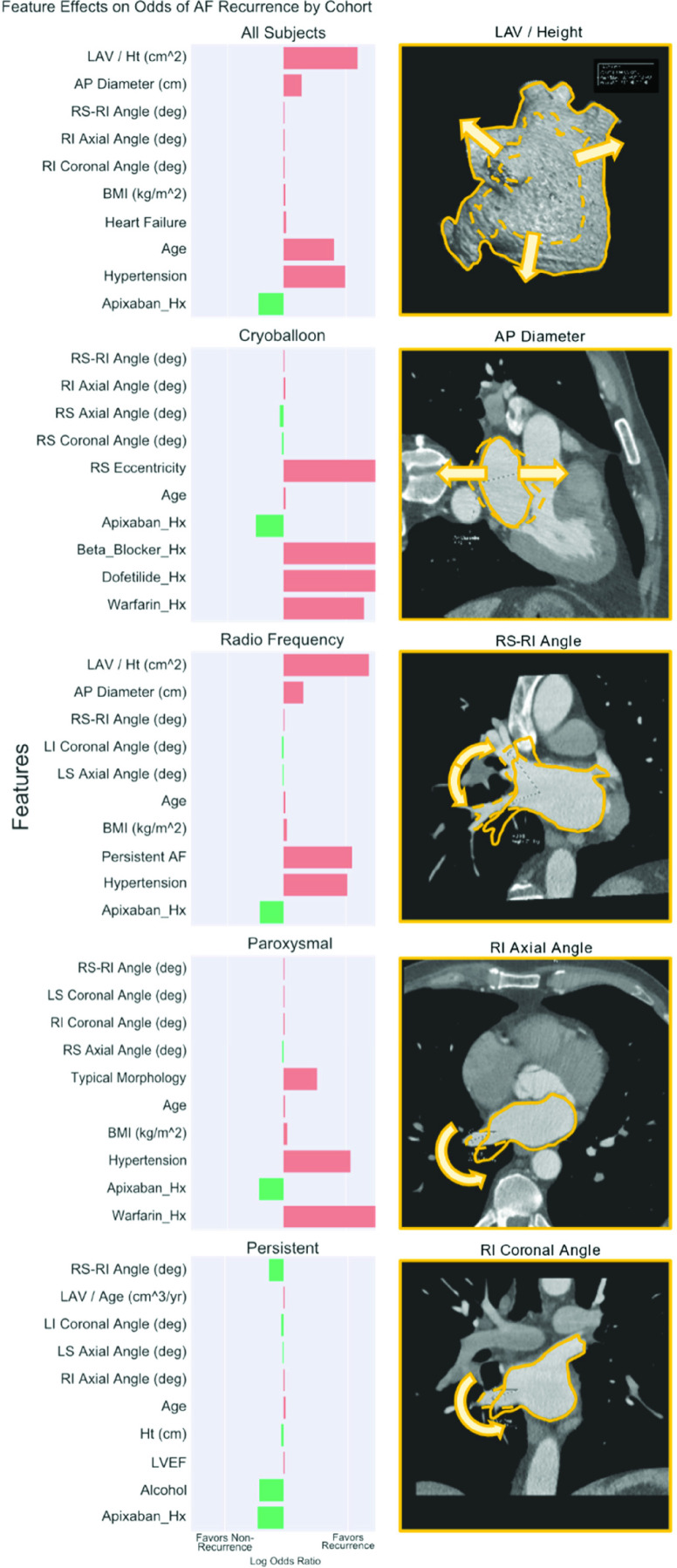
Left: Summary of 1-year post-PVI AF recurrence, Wilcoxon-selected, radiomic/clinical ( $\text{M}_{\mathrm {RC}}$) feature effect size by sub-cohort represented as log odds ratio in D_1_ subjects (n=150). Right: graphical representation of feature effects ( $\text{M}_{\mathrm {R}}$) for all subjects in D_1_.

The combination of Wilcoxon and LDA methods was identified as yielding superior performance for D_1_ subjects undergoing radiofrequency ablation, with radiomic-based models predicting post-PVI recurrence of AF with maximum AUC of 0.68 in independently validated D_2_ subjects. Logistic regression on most distinguishing features for post-PVI recurrence was performed on D_1_ subjects to gain intuition into the role of features towards predicting recurrence. Table 2 contains a summary of regression coefficients for all subjects included. For subjects undergoing radio-frequency ablation, a trend towards greater odds of recurrence was observed with greater right carina angle (OR 1.03, CI95 [1.00, 1.07]), as well as increased left atrial anterior-posterior diameter (OR 1.59, CI95 [0.99, 2.56]), increased left atrial volume normalized to patient height (OR 4.91, CI95 [0.97, 24.89]), and decreased left inferior and left superior pulmonary vein angles on the coronal (OR 0.96, CI95 [0.92, 1.00]) and axial (OR 0.98, CI95 [0.95, 1.01]) planes, respectively. These radiomic features are summarized graphically in Figure 5.

**TABLE 2:** Summary of Logistic Regression on Post-PVI Recurrence by Cohort for Features Selected by Wilcoxon Method for D_1_ in Red

Features	ALL SUBJECTS		CRYOBALLOON		RADIO-FREQUENCY		PAROXYSMAL		PERSISTENT	
	OR (CI_95_)	p-Value	OR (CI_95_)	p-Value	OR (CI_95_)	p-Value	OR (CI_95_)	p-Value	OR (CI_95_)	p-Value
LAV / Age (cm^3/yr)	0.92 (0.59, 1.45)	0.815	0.47 (0.14, 1.56)	0.363	1.02 (0.62, 1.68)	1.000	0.81 (0.39, 1.67)	0.705	0.58 (0.27, 1.21)	0.362
LAV / Ht (cm^2)	3.57 (0.87, 14.67)	0.148	0.75 (0.03, 19.13)	0.860	4.91 (0.97, 24.89)	1.000	2.12 (0.18, 25.31)	0.705	1.58 (0.16, 15.89)	0.836
AP Diameter (cm)	1.54 (1.03, 2.32)	0.084	1.37 (0.60, 3.11)	0.595	1.59 (0.99, 2.56)	1.000	1.33 (0.71, 2.49)	0.556	1.27 (0.58, 2.80)	0.730
RS-RI Angle (deg)	1.03 (1.01, 1.06)	0.039	1.03 (0.99, 1.07)	0.286	1.03 (1.00, 1.07)	1.000	1.03 (0.99, 1.06)	0.342	1.04 (1.00, 1.08)	0.286
LI Coronal Angle (deg)	0.98 (0.95, 1.01)	0.275	1.01 (0.95, 1.07)	0.834	0.96 (0.92, 1.00)	1.000	1.00 (0.96, 1.04)	1.000	0.94 (0.88, 1.00)	0.255
LS Axial Angle (deg)	0.99 (0.96, 1.01)	0.492	1.01 (0.97, 1.06)	0.671	0.98 (0.95, 1.01)	1.000	1.00 (0.97, 1.03)	1.000	0.98 (0.94, 1.02)	0.559
LS Coronal Angle (deg)	1.01 (0.99, 1.04)	0.325	1.03 (0.99, 1.08)	0.315	1.01 (0.98, 1.03)	0.965	1.03 (1.00, 1.06)	0.329	1.00 (0.97, 1.04)	0.924
RI Axial Angle (deg)	1.04 (1.00, 1.07)	0.078	1.06 (0.99, 1.13)	0.286	1.03 (0.99, 1.07)	1.000	1.03 (0.98, 1.08)	0.494	1.04 (1.00, 1.09)	0.286
RI Coronal Angle (deg)	1.04 (1.01, 1.07)	0.059	1.05 (0.99, 1.11)	0.286	1.04 (1.00, 1.08)	0.767	1.04 (1.00, 1.09)	0.238	1.03 (0.98, 1.08)	0.533
RS Axial Angle (deg)	1.00 (0.96, 1.03)	0.905	0.90 (0.83, 0.99)	0.131	1.02 (0.98, 1.07)	1.000	0.97 (0.92, 1.02)	0.494	1.02 (0.97, 1.08)	0.564
RS Coronal Angle (deg)	0.99 (0.96, 1.02)	0.700	0.96 (0.89, 1.02)	0.362	1.01 (0.97, 1.05)	1.000	0.98 (0.93, 1.02)	0.506	1.01 (0.96, 1.06)	0.836
RS Eccentricity	0.99 (0.14, 6.78)	0.988	7.50 (0.17, 326.98)	0.410	0.41 (0.04, 4.23)	0.200	1.51 (0.12, 18.61)	0.849	0.63 (0.03, 15.66)	0.868
Typical Morphology	1.34 (0.68, 2.62)	0.492	1.16 (0.32, 4.21)	0.860	1.43 (0.65, 3.15)	0.965	2.00 (0.85, 4.69)	0.342	0.34 (0.08, 1.41)	0.362
Age	1.06 (1.02, 1.11)	0.017	1.07 (0.98, 1.16)	0.286	1.06 (1.02, 1.11)	0.965	1.05 (1.00, 1.10)	0.238	1.07 (1.01, 1.14)	0.238
BMI (kg/m^2)	1.09 (1.02, 1.16)	0.033	1.02 (0.89, 1.17)	0.834	1.11 (1.03, 1.19)	1.000	1.12 (1.01, 1.23)	0.171	1.04 (0.95, 1.14)	0.564
Ht (cm)	0.97 (0.94, 1.00)	0.160	0.97 (0.91, 1.03)	0.401	0.98 (0.94, 1.01)	1.000	0.99 (0.95, 1.03)	0.752	0.94 (0.88, 1.00)	0.238
LVEF	1.02 (0.98, 1.06)	0.358	1.07 (0.99, 1.16)	0.286	1.00 (0.96, 1.05)	1.000	1.03 (0.97, 1.09)	0.506	1.04 (0.98, 1.10)	0.373
Persistent AF	2.11 (1.08, 4.13)	0.078	0.61 (0.15, 2.51)	0.616	3.06 (1.38, 6.80)	1.000	inf (0.00, inf)	1.000	inf (0.00, inf)	1.000
Alcohol	0.49 (0.24, 1.01)	0.108	0.41 (0.10, 1.66)	0.363	0.53 (0.23, 1.21)	0.800	0.61 (0.24, 1.57)	0.506	0.30 (0.09, 0.94)	0.238
Heart Failure	2.50 (1.27, 4.93)	0.033	2.06 (0.58, 7.29)	0.401	2.71 (1.21, 6.08)	1.000	1.84 (0.70, 4.84)	0.486	2.50 (0.82, 7.60)	0.331
Hypertension	2.83 (1.46, 5.49)	0.017	3.05 (0.83, 11.30)	0.286	2.89 (1.32, 6.33)	0.800	2.98 (1.27, 6.99)	0.100	2.18 (0.73, 6.55)	0.373
Apixaban_Hx	0.27 (0.13, 0.53)	<0.001	0.19 (0.05, 0.72)	0.131	0.31 (0.14, 0.70)	0.425	0.29 (0.12, 0.70)	0.075	0.25 (0.08, 0.78)	0.238
Beta_Blocker_Hx	1.06 (0.55, 2.02)	0.905	9.27 (1.73, 49.66)	0.131	0.58 (0.27, 1.25)	0.965	1.34 (0.58, 3.08)	0.682	0.62 (0.20, 1.91)	0.564
Dofetilide_Hx	1.88 (0.89, 3.97)	0.160	7.64 (1.38, 42.33)	0.131	1.20 (0.51, 2.82)	1.000	1.84 (0.70, 4.84)	0.486	1.77 (0.53, 5.99)	0.564
Warfarin_Hx	3.24 (1.38, 7.60)	0.033	4.28 (1.05, 17.42)	0.215	3.13 (1.04, 9.44)	0.965	6.60 (1.97, 22.09)	0.050	1.18 (0.34, 4.12)	0.868

OR = Odds Ratio (Unit OR for continuous variables); (CI_95_) = 95% Confidence Interval; p-Values corrected for multiple comparisons with FDR method AP = Anterior Posterior; LAV = Left Atrial Volume; LI =Left Inferior; LS = Left Superior; RI = Right Inferior; RS = Right Superior; PV= Pulmonary Vein; Hx = History; Ht = Height; LVEF = Left Ventricular Ejection Fraction; AF = Atrial Fibrillation

### B. Experiment 2: Clinical Features in Prediction of AF Recurrence Post-Ablation

The objective of this experiment was to demonstrate that clinical history predicts post-ablation recurrence of AF. Three clinical features were consistently identified in the top five measurements for D_1_ subjects, including age, BMI, and history of Apixaban use. Classifier models ( $\text{M}_{\mathrm {C}}$) for post-PVI recurrence trained on clinical features alone with radio-frequency ablation subjects resulted in a maximum AUC of 0.63 with the combination of Wilcoxon and LDA methods in the independent D_2_ cohort. Greater odds of recurrence were associated with greater age (OR 1.06, CI95 [1.02, 1.11]) and BMI (OR 1.11, CI95 [1.03, 1.19]), as well as diagnosis of hypertension (OR 2.89, CI95 [1.32, 6.33]) and persistent atrial fibrillation (OR 3.06, CI95 [1.38, 6.80]). History of Apixaban use was associated with reduced odds of post-PVI recurrence (OR 0.31, CI95 [0.14, 0.70]). Figure 5 graphically summarizes the effect sizes for radiomic and clinical features by subcohort.

### C. Experiment 3: Combination of Radiomic and Clinical Features in Prediction of AF Recurrence Post-Ablation

**FIGURE 6.**
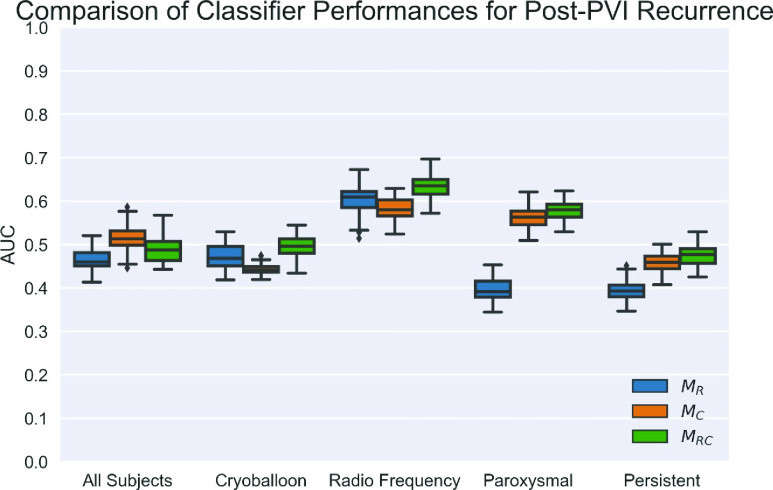
Comparison of LDA model performances using Wilcoxon-selected features by feature type and cohort subset on D_2_ reveals a trend for improved model performance combining radiomic and clinical features ( $\text{M}_{\mathrm {RC}}$), with best performance in subjects undergoing radio-frequency ablation. Radiomic- based models ( $\text{M}_{\mathrm {R}}$) were unable to reliably predict recurrence in patients with persistent AF.

The objective of this experiment was to evaluate whether combining clinical history with pulmonary vein and atrium morphology improves prediction of post-ablation recurrence of AF compared to each feature set alone. Classifier models ( $\text{M}_{\mathrm {RC}}$) trained simultaneously on D_1_ radio-frequency ablation subjects with the radiomic and clinical features identified in experiments 1 and 2 resulted in improved prediction of post-PVI recurrence (maximum AUC of 0.70) using the Wilcoxon and LDA methods in D_2_ subjects (Figure 6).

## IV. Discussion

Atrial fibrillation is the most common sustained arrhythmia [1], [2] and increases the risk of stroke and death [5]. Although recurrence rates for pulmonary vein isolation are high [7], identifying patients likely to have a successful outcome is challenging. Clinical factors such as age, gender, BMI, and AF type have modest success in predicting post-PVI recurrence [29], [30] and imaging-based features such as left atrium size [17], [18] and fibrosis scoring [20] show promise, though radiomic features of recurrence have not been extensively interrogated. Given these findings, we hypothesized that as yet uninterrogated features of cardiac and pulmonary venous anatomy are associated with and predictive of AF recurrence. In this work, we presented new radiomic features relating to PV-atrial angles and left atrium size and evaluated these features in conjunction with clinical features (e.g., Age, BMI, gender, cardiac history) to predict likelihood of post-PVI recurrence of AF. We also observed that radiomic features associated with post-PVI recurrence varied by AF type (see supplemental materials), supporting results from previous studies [30], [31].

Radiomic features of recurrence included greater right carina angle, greater left atrial anterior-posterior diameter, greater LA volume normalized to height, and greater angle of the right inferior PV on the coronal and axial planes. Our findings associating increased atrial size with recurrence are consistent with prior observations [17], [32], [33] and recent left atrial shape-based metrics that predict recurrence with AUC 0.71 [31]. This consistency with prior work, in conjunction with the conservation of selected features across several methods in our study, suggest a likely reproducibility of radiomic features in other populations, and we intend to study this next [34], [35]. To our knowledge, LA volume normalized to height has not been previously evaluated for recurrence of AF, although it is consistent with the known association of both increased LA volume and greater height with incidence AF [36]. The observed importance of PV angles, such as the right inferior PV, builds upon prior observations that PV angles differ between patients with AF and non-AF controls [12], and likely represents technically challenging anatomy during catheterization though anatomy as risk factors for unfavorable AF substrate might also be considered. Right carina angle is a new feature for recurrence which we constructed based on our hypothesis that PV angle on standard imaging planes may be influenced by cardiac positional variation within the chest (e.g., true anatomic or temporary positional variation). Other reported radiomic features, such as left atrial fibrosis assessed on MRI, predict post-ablation recurrence when combined with clinical and shape features (AUC = 0.72), though require non-standard MRI sequences and processing and have not yet been validated on an independent cohort [16]. The CT- based methods we present here are independently validated (AUC = 0.70 for radio-frequency ablation subjects) and are compatible with less expensive, standard imaging modalities and tools already integrated in existing clinical workflow. Combination of these approaches in the future may further improve prediction of recurrence and may be obtained using a single imaging modality.

Cardiovascular risk factors have been associated with left ventricular morphometry [37], and models combining clinical and radiomic features such as CAAP-AF predict recurrence after cryoballoon ablation (AUC = 0.71) [29], [30]. Our work improves upon these studies through use of an independent validation cohort with more typical rates of AF recurrence (33% compared to 8%), achieving comparable performance (AUC max = 0.70 in radio-frequency ablation subjects) while utilizing fewer clinical parameters, including: age, BMI, and medication history. Our observation associating apixaban use with reduced likelihood of recurrence and warfarin use with increased likelihood may be a proxy for factors such as disease state rather than a potential causative agent, and additional validation in larger cohorts is required.

Limitations of this work include small sample size and lack of multi-center data. Models trained on the entire cohort (i.e., “All Subjects”) were not improved by combining radiomic and clinical features, though this may have resulted from a validation cohort predating cryoballoon-use, as supported by improved performance when controlling for catheter-type in the training set (D_1_). This may also limit inferences regarding radiomic features to subjects undergoing radiofrequency ablation as features appear to vary by catheter type (Figure 3). Another limitation was reliance on manual image annotation and semi-automated PV segmentation without investigating the variance among multiple readers and its impact on extracted radiomic features. Findings are strengthened by independent validation, though would be further strengthened by additional prospective validation. Inferring the effect of varying individual features is challenging using machine learning methods, though the logistic regression methods described above may lend insight to clinicians in their decision-making process.

In this study, we presented new radiomic features relating to left atrium size and right-sided pulmonary vein angles, and in conjunction with clinical features, these radiomic features were found to be associated with risk of post-ablation recurrence of AF within three months to one year in patients without valvular disease or prior cardiac surgery. These were found to be a function of type of ablation and type of AF and best predict recurrence in subjects who underwent radio-frequency ablation, but require additional multi-site data with RF and cryoballoon ablation to validate the findings from this study.
